# The risk perception of COVID-19 and practice of precautionary measures amongst healthcare workers in the National Health Insurance Scheme Clinic of a tertiary hospital in Nigeria

**DOI:** 10.11604/pamj.2021.38.73.27427

**Published:** 2021-01-21

**Authors:** Osahon Enabulele, Aihevba Esther

**Affiliations:** 1 Department of Family Medicine, University of Benin Teaching Hospital, Ugbowo, Benin City, Edo State, Nigeria,; 2 Department of Family Medicine, University of Benin, Benin City, Edo State, Nigeria

**Keywords:** COVID-19, healthcare worker, National Health Insurance Scheme, precautionary measure, risk perception

## Abstract

**Introduction:**

the novel coronavirus (SARS-CoV-2), the causative virus for coronavirus disease 2019 (COVID-19), was identified following the report of a cluster of cases of viral (atypical) pneumonia in Wuhan City of China. Healthcare workers are at high risk of contracting the infection from COVID-19 patients and also spreading it unknowingly to their families, especially if they do not take adequate precautionary measures. This study assessed the risk perception of COVID-19 and practice of precautionary measures against its spread amongst healthcare workers practicing in the National Health Insurance Scheme (NHIS) Clinic of a tertiary hospital in Nigeria.

**Methods:**

this was a descriptive cross-sectional study conducted amongst healthcare workers in the National Health Insurance Scheme (NHIS) Clinic of a tertiary hospital in Nigeria. It employed the use of a pre-tested semi-structured questionnaire to obtain data from the participants. Data analysis was done using the IBM SPSS statistics version 22.0 (Chicago, IL, USA) statistical software.

**Results:**

there were 49 study participants with all of them aware of COVID-19. Only 11(22.4%) respondents reported receiving training on infection prevention and control against COVID-19. Most of them received training from their workplace/hospital (12.2%), while 10.2% were trained via webinars. More of the respondents had moderate risk perception (n=17, 34.7%) while a majority of them had good practice of precautionary measures against COVID-19 (n=28, 57.1%). **Conclusion:** most of the study participants had moderate risk perception and good practice of precautionary measures. Risk perception was not a significant predictor of practice of precautionary measures.

## Introduction

The novel coronavirus was identified and named SARS-CoV-2 following the report of a cluster of cases of viral (atypical) pneumonia in Wuhan, Hubei Province of China [1]. This report was made to the World Health Organization (WHO) by the Wuhan Municipal Health Commission, China, on the 31^st^ of December, 2019 [1]. The disease associated with this virus (SARS-CoV-2) was named coronavirus disease 2019 (COVID-19) by the WHO on 11^th^ of February, 2020 [1]. COVID-19 was declared by WHO as a public health emergency of international concern (PHEIC) on 30^th^ January 2020 [2] and on 11^th^ March 2020, WHO declared COVID-19 a pandemic disease [3]. COVID-19 is highly infectious and majorly transmitted through respiratory droplets produced from an infected person while sneezing or coughing [4].

Healthcare workers (HCWs) are extremely strained during the course of any pandemic (such as the COVID-19 pandemic) because of their role as key players in the response to a pandemic [5]. Healthcare workers are at high risk of not only contracting the infection from COVID-19 patients but also spreading it unknowingly, especially if they do not take adequate precautionary measures [6]. Since the emergence of COVID-19, several HCWs have been infected at their workplaces. Others have, unfortunately lost their lives. Because healthcare-associated infection of healthcare workers is a major problem, the Centre for Disease Control (CDC) recommends that healthcare workers use personal protective equipment (PPE) and implement standard contact and airborne precautions, including eye protection. It is recommended that healthcare workers should wear a gown, gloves and either an N95 respirator plus a face shield or goggles or a powered, air-purifying respirator [7].

Avoiding cross-infection from patients along with effective care delivery can be achieved if the healthcare workers (including physicians, pharmacists, nurses and other medical staff) have a positive attitude towards the disease and better precautionary practices against the spread of COVID-19 [6]. Recognizing the influence of risk perception of individuals (including HCWs) on their practice behaviours, this study sought to assess the risk perception of COVID-19 and practice of recommended precautionary measures against its spread amongst healthcare workers employed in the National Health Insurance Scheme (NHIS) Clinic of a tertiary hospital. This was with a view to obtaining insights on the subject matter to positively influence hospital policy on workplace safety, as well as the practice behaviours of the healthcare workers.

## Methods

This was a descriptive cross-sectional study conducted in the month of June, 2020, during the COVID-19 pandemic. The study was conducted amongst healthcare workers (medical doctors, nurses, pharmacists, medical laboratory scientists and other allied health professionals and health workers) working in the National Health Insurance Scheme (NHIS) Clinic of the University of Benin Teaching Hospital (UBTH), Benin City, Edo State, Nigeria. The National Health Insurance Scheme (NHIS) Clinic in the study setting is an insurance-based outpatient primary care/first contact clinic that offers care to enrollees on Nigeria´s contributory health insurance scheme, known as the National Health Insurance Scheme (NHIS). The fact that the healthcare workers in this clinic are usually the first to make contact with the enrollees, increases their risk of exposure to COVID-19.

A pretested semi-structured questionnaire was used to obtain data on the socio-demographic characteristics (age, gender, marital status etc.) of the study participants. It was also used to obtain data on their risk perception of coronavirus disease (COVID-19) and their practice of precautionary measures against the spread of SARS-CoV-2. Ethical clearance was obtained from the Health Research Ethics Committee of the University of Benin Teaching Hospital. Informed written and voluntary consent was obtained from all participants after the purpose, procedure and benefits of the study were explained. All the data obtained from the participants were kept strictly confidential and used solely for the purpose of the research study.

Risk perception was assessed using the question, ‘how will you assess your risk of contracting COVID-19 in your workplace?´, to which respondents were to reply ‘low´, ‘moderate´, ‘high´ or ‘not sure´. Practice of precautionary measures against the spread of SARS-CoV-2 was assessed using 17 yes or no questions. Each correct response was scored “1” and each wrong response “0”. Scores ranged from 0 to 17. Participants´ practice of precautionary measures against COVID-19 were classified as poor (≤9) or good (≥10). Regression analysis was performed to identify the factors associated with risk perception and practice of precautionary measures against COVID-19 infection. P≤0.05 was considered statistically significant.

All data obtained from the study were validated, coded and analysed using the IBM SPSS statistics version 22.0 (Chicago, IL, USA) statistical software. Descriptive statistics such as mean and standard deviation (SD) were used to present numerical data, while frequency (number) and percent were used to present categorical (qualitative) data. Chi-square test was used to compare qualitative variables. Regression analyses were performed to identify the factors associated with risk perception and practice of precautionary measures against COVID-19 infection. P≤0.05 was considered statistically significant.

## Results

**Figure 1:**
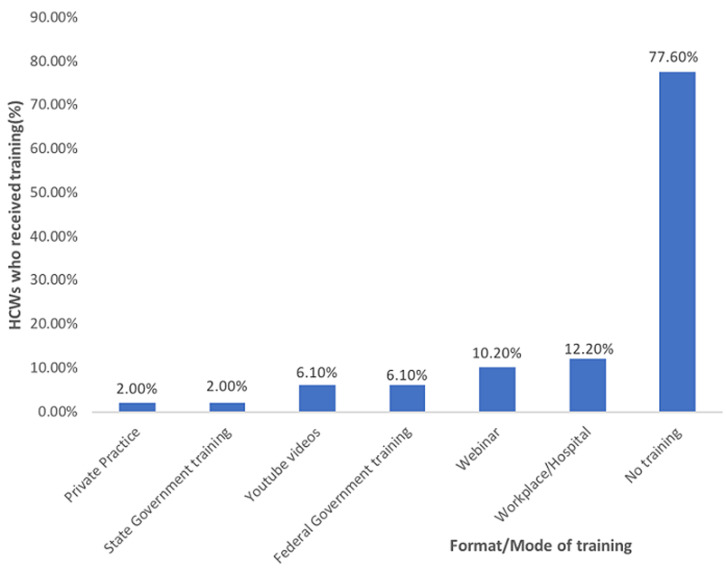
sources of training on infection prevention and control against COVID-19

**Sociodemographic characteristics of respondents:** forty-nine (49) healthcare workers (HCWs) in the National Health Insurance Scheme (NHIS) Clinic of UBTH participated in this study out of a total of 50 healthcare workers employed in the clinic, giving a response rate of 98%. There was a slight female preponderance (55.1%) with a male-female ratio of 1: 1.2. The age of the respondents ranged from 23 to 58 years with a mean age of 35.5 ± 8.6 years. The age groups of <30 years and 31-40 years were equally represented (36.7%). Majority of the respondents were married (63.3%), had tertiary education (87.8%) and had practiced in their current place of work for less than 10 years (85.7%). Pharmacists were the most prevalent healthcare workers (28.6%) followed by medical doctors (26.5%) (Table 1). All the participants in this study were aware of COVID-19. However, only 11(22.4%) respondents reported that they had received training on infection prevention and control against COVID-19. Most of them received training from their workplace/hospital (12.2%), while 10.2% were trained via webinars (Figure 1).

**Table 1:** socio-demographic characteristics of respondents

Socio-demographic characteristic	Frequency (N=49)	Percentage (%)
**Age (years)**		
≤30	18	36.7
31-40	18	36.7
>40	13	26.6
**Gender**		
Male	22	44.9
Female	27	55.1
**Marital status**		
Single	17	34.7
Married	32	65.3
**Occupation**		
Medical doctor	13	26.5
Nurse	10	20.4
Pharmacist	14	28.6
Others	12	24.5
**Level of education**		
Secondary	6	12.2
Tertiary	43	87.8
**Length of practice (years)**		
<10	42	85.7
>10	7	14.3

Others: other healthcare workers i.e. records clerks, chew, medical laboratory scientists, technicians

**Figure 2:**
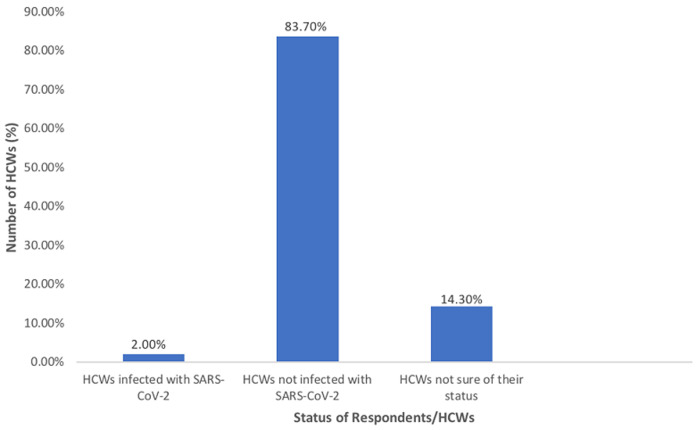
National Health Insurance Scheme healthcare workers infected by COVID-19

**Risk perception and attitude towards COVID-19:** a higher proportion of the respondents (83.7%) were afraid of contracting the disease, while 12.2% were not afraid of being infected and 4.1% were not sure. Only 6.1% of the respondents had screening done and all reported it was part of routine screening. Most of the respondents (83.7%) had not been infected with the novel coronavirus (SARS-CoV-2), however, a minority (14.3%) were not sure and only 2.0% had previously been infected (Figure 2). Majority of the respondents (61.2%) reported they do not feel safe and secure at work. Only 24.5% of the respondents agreed they felt safe and secure at work, while 14.3% were uncertain if they were safe and secure.

More (34.7%) of the respondents reported they had moderate susceptibility to contracting COVID-19. This was followed by 13(26.5%) of the respondents who stated they had low susceptibility and were not sure of their susceptibility, while 6(12.2%) reported they had high susceptibility to COVID-19. Over half of the participants (59.2%) never felt like stopping work for fear of contracting COVID-19, while 36.7% of them stated that they felt like stopping work. A vast majority (87.8%) agreed that use of personal protective equipment (PPE) can reduce the risk of contracting COVID-19 while 10.2% of them didn´t think so and 2.0% were not sure. Of the 87.8% who stated PPE can reduce the risk of contracting COVID-19, a majority (49.0%) agreed to a reduction in contracting the infection by 50-75%, while a reduction by 76-100%, 10-49% and <10% were reported by 24.5%, 8.2% and 2.0% of the respondents respectively. A higher proportion of the respondents (67.3%) stated they use PPE at place of work, while 32.7% said they didn´t use PPE.

A total of 43 (87.8%) participants stated there have been reported COVID-19 cases at work, 12.5% were not sure, while 2.0% stated there was no case of COVID-19 at place of work. Conversely, only 8.2% reported contact with a confirmed case of COVID-19, while 63.3% had no contact and 28.6% were not sure. All the respondents with contacts with confirmed cases stated contacts were at place of work. More respondents in this study (46.9%) stated that they had contact with a suspected case of COVID-19, whereas 30.6% reported no contact and 22.4% of them were not sure of contact with suspected cases. There were more reported contacts with suspected cases at place of work (42.9%), while 2.0% had contact with a suspected case at home. Interestingly, 18 (36.7%) of the respondents with contact with confirmed or suspected cases stated they continued with work routine, while 5(10.2%) isolated after informing the hospital management.

**Distribution of respondents´ risk perception of COVID-19 by sociodemographic characteristics:** more respondents had moderate risk perception (34.7%), while 26.5% had low risk perception. Only 12.2% had high risk perception of COVID-19, however, 13(26.5%) were not sure of their risk perception. The relationship between risk perception and occupation of respondents was statistically significant (p=0.033). More nurses (20.0%) had high risk perception compared to other occupations, and a lower proportion of doctors (14.3%) had low risk perception (Table 2).

**Table 2:** distribution of respondents' risk perception of COVID-19 by sociodemographic characteristics

Sociodemographic characteristics	Risk perception categories				Chi square/p value
	Low N=13;(26.5%)	Moderate N=17;(34.7%)	High N=6;(12.2%)	Not sure N=13;(26.5%)	
**Age (years)**					
≤30	8 (44.4)	7 (38.9)	0 (0.0)	3 (16.7)	**9.643; p=0.14
31-40	2 (11.1)	7 (38.9)	4 (22.2)	5 (27.8)	
>40	3 (23.1)	3 (23.1)	2 (14.30	5 (38.5)	
**Gender**					
Male	8 (36.4)	10 (45.5)	1 (4.5)	3 (13.6)	**7.223; p=0.065
Female	5 (18.5)	7 (25.9)	5 (18.5)	10 (37.0)	
**Marital status**					
Single	5 (29.4)	9 (52.9)	0 (0.0)	3 (17.6)	**6.542; p=0.088
Married	8 (25.0)	8 (25.0)	6 (18.8)	10 (31.3)	
**Occupation**					
Medical doctor	2 (15.4)	7 (53.8)	1 (7.7)	3 (23.4)	**9.078; p=0.022*
Nurse	2 (20.0)	5 (50.0)	2 (20.0)	1 (10.0)	
Pharmacist	6 (42.9)	3 (21.4)	1 (7.1)	4 (28.6)	
Others	3 (27.3)	1 (9.1)	2 (18.2)	5 (45.5)	
**Level of education**					
Secondary	1 (16.7)	1 (16.7)	1 (16.7)	3 (50.0)	**2.420; p=0.490
Tertiary	12 (27.9)	16 (37.2)	5 (11.6)	10 (23.3)	
**Length of practice**					
≤10 years	11 (26.2)	14 (33.3)	5 (11.9)	12 (28.6)	**0.99; p=0.94
>10 years	2 (28.6)	3 (42.9)	1 (14.3)	1 (14.3)	

Others: other healthcare workers i.e. records clerks, chews, medical laboratory scientists, technicians; *statistically significant; **Fisher's exact

**Respondents´ practice of precautionary measures against COVID-19:** in this study, majority (57.1%) of the health workers had good practice of precautionary measures against COVID-19, while 42.9% had poor practice. A higher proportion of the respondents (59.2%) stated they had necessary preventive measures in their place of work. Over 90% of respondents agreed to the use of facemask (93.9%) and regular hand washing with soap and water (93.9%) in their workplace as preventive measures against COVID-19, while 89.8% agreed to the use of alcohol-based hand sanitizers. However, 6.1% of the respondents stated that herbal supplements were used in their workplace. Most health workers reported that they always follow recommendations from health authorities to prevent spread of COVID-19 (44.9%) while 6.1% stated they don´t follow the recommendations. The most commonly used preventive measures by participants were washing of hands regularly with soap and water for 20 seconds (85.7%), avoid touching eyes, nose and mouth with unwashed hands (83.7%), wearing facemask at work and in public (77.6%) and the use of alcohol-based hand sanitizer (73.5%). The least reported preventive measure was use of herbal and traditional medicine remedies (8.2%).

The relationship between respondents´ practice of precautionary measures and their sociodemographic characteristics is shown in Table 3. Medical doctors had a significantly higher proportion of good practice of precautionary measures against COVID-19 (92.3%) compared to other occupations (p=0.022). Occupation and level of education were significantly associated with practice of precautionary measures against COVID-19 (OR= 0.253; 95% CI: 0.089-0.719; OR= 0.209; 95% CI: -0.120-0.371 respectively).

**Table 3:** distribution of respondents practice of precautionary measures against COVID-19 by their sociodemographic characteristics

Sociodemographic characteristics	Practice of precautionary measures^a^		Test statistic/p value	OR (95% confidence interval)	P value
	Good N=28 (57.1%)	Poor N=21 (42.9%)			
**Age (years)**					
≤30	11 (61.1)	7 (38.9)	**5.342; p=0.07	0.925 (0.825-1.037)	0.07
31-40	13 (72.2)	5 (27.8)			
>40	4 (30.8)	9 (69.2)			
**Gender**					
Male	12 (54.5)	10 (45.5)	χ2=1.110; p=0.740	0.939 (0.182-4.830)	0.940
Female	16 (59.3)	11 (40.7)			
**Marital status**					
Single	12 (70.6)	5 (29.4)	χ2=1.922; p=0.166	1.168 (0.150-9.089)	0.882
Married	16 (50.0)	16 (50.0)			
**Occupation**					
Medical doctor	12 (92.3)	1 (7.7)	**12.306; p=0.006*	0.253 (0.089-0.719)	0.010*
Nurse	6 (60.0)	4 (40.0)			
Pharmacist	7 (50.0)	7 (50.0)			
Others	3 (25.0)	9 (75.0)			
**Level of education**					
Secondary	3 (50.0)	3 (50.0)	**0.142; p=0.706	0.209 (-0.120-0.371)	0.024*
Tertiary	25 (58.1)	18 (41.9)			
**Length of practice**					
≤10 years	25 (59.5)	17 (40.5)	**3.969; p=0.68	1.010 (0.871-1.148)	0.998
>10 years	3 (42.9)	4 (57.1)			

a: total score ranged from 0 to 17, a score of ≤9 was set for poor and ≥10 set for good practice of precautionary measures against COVID-19; others: other health care workers i.e. records clerks, chews, medical laboratory scientists, technicians; *statistically significant; **Fisher's exact

## Discussion

The mean age and most prevalent age group recorded in this study is similar to that reported in a study done in south-south, Nigeria, where the mean age of respondents was 33.6 ± 9.3 years, with majority of respondents in the age group, 31-40 years [8]. Majority of the health workers in this study were females and this was also seen in similar studies [8,9]. The level of education recorded in this study was secondary and tertiary level of education. This is in consonance with the minimum educational requirement of secondary school leaving certificate for gainful employment in federal government organizations in Nigeria. Pharmacists made up a higher percentage of respondents in this study. This is similar to a study done in Islamabad, Pakistan [10]. Conversely, in studies done in Vietnam, China and Ethiopia, majority of the respondents were nurses [5,11,12], while doctors made up majority of the respondents in a study in Libya [13].

All the respondents in this study were aware of COVID-19 agreeing with findings in a similar study [9]. Most of the respondents did not feel safe in the workplace and were afraid of contracting COVID-19. Similar finding was observed in China [12]. This fear is understandable, as HCWs are more likely to be in contact with infected persons than other members of the society. However, only 8.2% had been in contact with confirmed cases, while 46.9% had been in contact with suspected cases. This study finding may also have been due to inadequate supplies of personal protective equipment (PPEs) and the non-exposure of most of the respondents to training on infection prevention and control programmes as reported in this study.

On the respondents´ risk perception of COVID-19, while majority of the respondents (34.7%) had moderate-risk perception, 26.5% had low-risk perception and 12.2% had high-risk perception. This is similar to a study done in Portugal where 54.9% of the respondents had moderate-risk perception [14]. Health care workers constitute the frontline defence in combating the scourge of COVID-19. If the perceived risk of contracting the disease is high among health care workers, they may begin to reduce their number of work hours and workdays for fear of being infected, thus reducing the number of healthcare workers needed to deliver healthcare services in this crucial time. Only about a third of HCWs in the present study felt like stopping work at the time of this study. It was also shown in the present study that 2% of the HCWs had contracted COVID-19 as at the time of the study. This finding was similar to findings in France where there was a 2.2% prevalence of COVID-19 among healthcare workers, with a range of 0-3.6%, depending on the region [15]. Health care workers, especially primary care workers have high infection rate owing to treating patients with COVID-19 and can therefore increase the spread of the disease to the public. This can only be mitigated by the proper practice of precautionary measures.

Regular hand washing with soap and water, wearing face mask at work or in public and use of alcohol-based hand sanitizers are common preventive measures against COVID-19 reported by the respondents. These preventive measures were also reported among respondents in several studies [8,9,11]. In the present study, most respondents had good practice of precautionary measures against COVID-19. This is commendable as it can reduce the risk of nosocomial spread of the disease, as well as transmission of the infection by health care workers to their families. Among the respondents, medical doctors had better practice of precautionary measures than other cadres of healthcare workers (OR: 0.253, 95% CI: 0.089-0.719). A similar finding was reported in a study in Pakistan [10]. This may be an effect of their better knowledge of the disease. The result from this study showed that risk perception among healthcare workers did not affect their practice of precautionary measures. While one would have expected this to positively influence the practice behaviour of the respondents, the finding is not entirely surprising as it has been reported in other studies that individual perception of infectious illnesses such as COVID-19 may not be sufficient to influence the adoption of protective practices [16,17]. This finding underscores the need to regularly expose healthcare workers to health education/enlightenment and training programs/seminars on COVID-19, including infection prevention and control protocols/measures.

**Limitations:** the findings from this study may be ungeneralizable to the general population of healthcare workers in Nigeria. This is particularly on account of the fact that this study surveyed only healthcare workers in the National Health Insurance Scheme (NHIS) Clinic of only one of the tertiary hospitals in Nigeria, University of Benin Teaching Hospital (UBTH).

**Strength:** the study limitation, notwithstanding, this study has provided useful perspectives that can help reinforce workplace policies and training of healthcare workers, as well as aid further research on the study subject.

## Conclusion

The COVID-19 pandemic has challenged every aspect of humanity and healthcare workers have been at the fore front of combating the scourge. Most of the healthcare workers in this study had good practice of precautionary measures and moderate risk perception. However, about a third of them felt like stopping work for fear of contracting COVID-19. This can hinder effective delivery of healthcare services. It is therefore recommended that there should be sustained provision of personal protective equipment (PPE), indemnity and health insurance cover and a protective work environment for healthcare workers. Importantly, workplace safety policies and incentive packages should be implemented to boost healthcare workers´ confidence and their willingness to effectively provide optimal healthcare services, amidst this ravaging COVID-19 pandemic. It is also recommended that training programs and seminars should be regularly organized for healthcare workers (especially allied health workers) on infection prevention and control practices/protocols, to enable them carry out their professional duties with minimal risk to themselves and their families. Further studies, including multicenter studies, are also recommended.

### What is known about this topic

- Healthcare workers, particularly those who offer first contact care to patients, are at high risk of contracting the infection from COVID-19 patients;
- Infected healthcare workers can unknowingly spread COVID-19, especially if they are not trained on infection prevention and control measures, and therefore do not take adequate precautionary measures against the spread of the infection.

### What this study adds

- This study, the first of its kind in the study setting, uniquely explored the risk perception of COVID-19 and practice of precautionary measures against its spread amongst a cohort of healthcare workers employed in an insurance-based (National Health Insurance Scheme) clinic of a tertiary hospital. The study therefore provides useful insights and perspectives that can help reinforce workplace policies and training of healthcare workers, particularly the cohort of healthcare workers employed by Nigeria´s National Health Insurance Scheme Clinic;
- The findings from this study can be leveraged upon to further research on the study subject.
